# p53-upregulated-modulator-of-apoptosis (PUMA) deficiency affects food intake but does not impact on body weight or glucose homeostasis in diet-induced obesity.

**DOI:** 10.1038/srep23802

**Published:** 2016-04-01

**Authors:** Sara A. Litwak, Kim Loh, William J. Stanley, Evan G. Pappas, Jibran A. Wali, Claudia Selck, Andreas Strasser, Helen E. Thomas, Esteban N. Gurzov

**Affiliations:** 1 St Vincent’s Institute of Medical Research, Melbourne, Australia; 2 Department of Medicine, St. Vincent’s Hospital, The University of Melbourne, Melbourne, Australia; 3 The Walter and Eliza Hall Institute of Medical Research, Parkville, Australia; 4 Department of Medical Biology, The University of Melbourne, Melbourne, Australia

## Abstract

BCL-2 proteins have been implicated in the control of glucose homeostasis and metabolism in different cell types. Thus, the aim of this study was to determine the role of the pro-apoptotic BH3-only protein, p53-upregulated-modulator-of-apoptosis (PUMA), in metabolic changes mediated by diet-induced obesity, using PUMA deficient mice. At 10 weeks of age, knockout and wild type mice either continued consuming a low fat chow diet (6% fat), or were fed with a high fat diet (23% fat) for 14–17 weeks. We measured body composition, glucose and insulin tolerance, insulin response in peripheral tissues, energy expenditure, oxygen consumption, and respiratory exchange ratio *in vivo*. All these parameters were indistinguishable between wild type and knockout mice on chow diet and were modified equally by diet-induced obesity. Interestingly, we observed decreased food intake and ambulatory capacity of PUMA knockout mice on high fat diet. This was associated with increased adipocyte size and fasted leptin concentration in the blood. Our findings suggest that although PUMA is dispensable for glucose homeostasis in lean and obese mice, it can affect leptin levels and food intake during obesity.

The prevalence of obesity is increasing at an alarming rate worldwide^1^. It is a major cause of morbidity and mortality and is estimated to account for ~10% of healthcare costs in developed nations^2^. According to the world health organization (http://www.who.int), there are ~1.9 billion overweight adults [Body mass index (BMI) >25 kg/m^2^] worldwide of whom more than 600 million are obese (BMI>30 kg/m^2^). Obesity is a major risk factor for the development of insulin resistance that can lead to type 2 diabetes and severe complications such as cardiovascular disease, blindness and renal failure. It is clear that a diet rich in saturated fats and a sedentary lifestyle result in obesity^3^,^4^. However, the molecular pathways affected and proteins involved in the different tissues during fat accumulation and body weight gain are not well characterised^2^.

The BCL-2 family of proteins are regulators of the intrinsic apoptotic pathway^5^. There are three groups of proteins in the BCL-2 family: the pro-survival proteins (BCL-2, BCL-XL, MCL-1, BCL-W, A1/BFL1), the multi-BH domain pro-apoptotic proteins (BAX, BAK, BOK) and the pro-apoptotic BCL-2 homology 3 (BH3)-only proteins (BID, BIM, BAD, BMF, NOXA, DP5, BLK, and p53-upregulated-modulator-of-apoptosis (PUMA))^6^. The BH3-only proteins trigger apoptosis either by directly activating BAX/BAK or indirectly by binding to the pro-survival BCL-2 proteins thereby preventing their restraint of pro-apoptotic BAX/BAK.

We have previously demonstrated that the BH3-only protein PUMA is activated in pancreatic β-cells after exposure to saturated free fatty acids, high glucose concentrations, certain cytokines or chemical endoplasmic reticulum (ER) stressors, leading to the activation of BAX and β-cell death *in vitro*^7^,^8^,^9^,^10^. Deficiency of PUMA prevents BAX activation, mitochondrial cytochrome *c* release and caspase-3 cleavage in these settings, protecting β-cells from apoptosis. In addition, PUMA contributes to β-cell apoptosis in high fat fed Pdx1-deficient mice^11^. In hepatocytes, saturated free fatty acids induce cell death through BIM and PUMA upregulation^12^. Moreover, hepatosteatosis and hepatocellular carcinoma in liver-specific STAT5 knockout mice is associated with downregulation of BIM and PUMA^13^. Finally, BCL-2 proteins have been reported to regulate cell death in adipocytes during the development of obesity^14^,^15^.

Recent evidence suggests that BCL-2 proteins not only control apoptosis induction, but can also play an important role in glucose homeostasis and metabolism. For example, Bcl-XL overexpression decreases pancreatic β-cell insulin secretion^16^. This may be due to the fact that BCL-XL causes survival of aged β-cells with lesser secretory capacity. In addition, phosphorylation of the BH3-only protein BAD might activate glucokinase to control insulin release and hepatic gluconeogenesis in mice^17^,^18^. Moreover, BCL-2 proteins have been reported to regulate glucose metabolism through the pentose phosphate pathway^19^, mitochondrial activity^20^ or Ca^2+^ trafficking^21^.

While it is well accepted that *in vitro* PUMA has a role in apoptosis induction in cell types involved in metabolism, including β-cells and hepatocytes, its role in metabolism *in vivo* is unclear. It is also unknown whether, through its role as an apoptosis initiator, PUMA may affect the control of glucose homeostasis and metabolism in the development of obesity and insulin resistance. In the present work, we provide evidence that loss of PUMA influences circulating leptin levels and food intake but has no impact on glucose homeostasis in diet-induced obesity.

## Materials and Methods

### Mice

Mice were maintained at St. Vincent’s Institute animal care facility on a 12 h light-dark cycle in a temperature-controlled room and obtained food and water *ad libitum*. PUMA knockout mice were generated on a C57BL/6 background as previously described^22^. Male mice were kept on regular chow (20% protein, 6% fat and 3.2% crude fibre) or placed at 10 weeks of age on a high fat diet (SF04-027 Speciality Feeds, Perth, Western Australia) for 14–17 weeks. The nutritional composition of the high fat diet was 18.4% protein, 23.5% fat and 4.7% crude fibre. In this diet, 46% of total energy is from lipids, 20% of total energy from protein and the remainder from carbohydrates.

At the conclusion of the experiment, mice were euthanized by cervical dislocation and organs were obtained and their weight recorded. Tissues were snap frozen for Western Blot and real time RT-PCR analysis or formalin fixed for histological analysis. To examine insulin signalling, a subset of fasting animals were injected intraperitoneally (i.p.) with human insulin (0.65 mU/g, Actrapid, Novo Nordisk, Denmank) 10 min before organ retrieval.

All animal studies were conducted at St Vincent’s Institute (Melbourne, Australia) following the guidelines of the Institutional Animal Ethics Committee. Animal ethics was approved by the St Vincent’s Hospital Animal Ethics Committee and the experiments were carried out in accordance with the approved guidelines.

### Culture and *in vitro* treatment of mouse islets

Mouse islets were isolated using Collagenase P (Roche, Basel, Switzerland) and Histopaque-1077 density gradients (Sigma, St Louis, MO, USA) as previously described^10^. Islets were washed, hand-picked and cultured overnight at 37 °C in 5% CO_2_ in CMRL medium-1066 (Invitrogen) supplemented with 100 U/ml penicillin, 100 μg/mL streptomycin, 2 mmol/L glutamine and 10% FCS (JRH Biosciences, Lenexa, KS, USA).

### Histology analysis

Liver and adipose tissue (gonadal fat pads) were isolated from PUMA knockout or wild type male mice after 17 weeks of high fat feeding. The same region of the liver and fat pad was used for all animals to minimize variation. The samples were fixed in formalin, embedded in paraffin, cut into 5 μm sections, and stained with haematoxylin and eosin.

After 16 weeks of high fat feeding, PUMA knockout and wild type animals received a single injection of recombinant murine leptin (0.2 μg/g; Peprotech, Rocky Hill, NJ, USA) and after 30 min they were anesthetized and their brains perfused with saline and then 4% paraformaldehyde. Brains were postfixed in 4% PFA, then placed in 30% sucrose overnight and cut at 30 μm on a cryostat. Subsequently, immunohistochemistry was performed using antibodies against p-STAT3 (Cell Signaling, Danvers, MA). The numbers of p-STAT3-positive neurons in the arcuate nucleus within a constant and defined frame were counted using ImageJ software (National Institutes of Health).

### Real-time RT- PCR

RNA was extracted from liver and gonadal fat pads and prepared using the NucleoSpin RNA XS kit (Macherey Nagel, Düren, Germany). First-strand cDNA was prepared from 600 ng RNA using the High Capacity cDNA Reverse Transcription kit (Applied Biosystems, Foster City, CA, USA). cDNA was diluted (1:20) and real-time PCR was performed using the Roche LightCycler® 480 Instrument II (Corbett Research; Qiagen, Hilden, Germany) and the TaqMan PCR Master Mix (AmpliTaq Gold with GeneAmp kit; Applied Biosystems) in 20 μL reaction volumes. Data analyses were performed with the ddCT method using β-actin or 18S rRNA as an internal control. Results are represented as fold induction compared to control. TaqMan gene expression probes for mouse gluconeogenic, lipogenic and inflammatory genes (Applied Biosystems) are provided in Supp. Table 1.

### Western blot

Muscle, liver and white adipose tissue (gonadal) were lysed using RIPA buffer and total proteins were extracted and resolved by SDS-PAGE, transferred onto a nitrocellulose membrane and immunoblotted with anti-p-AKT, anti-AKT, anti-BCL2 and anti-BIM (Cell Signaling) antibodies^23^. The intensity values for the protein bands were corrected by the values of the housekeeping protein β-actin (Santa Cruz Biotechnology, CA) or α-tubulin (Sigma, St Louis, MO, USA) used as loading controls.

### Glucose, insulin and leptin tolerance test

Intraperitoneal glucose tolerance tests (ipGTT) were performed after a 6 h fast (2 g/kg dextrose) after 16 weeks on the chow and high fat fed groups as previously described^23^. Tail-knick blood samples were taken and glucose concentration measured with a standard glucometer (Accu-Check Performa, Roche) at 0, 30, 90 and 120 min after injection.

At week 17 on high fat or chow diet, insulin tolerance test was performed after a 4 h fast by administering human recombinant insulin (0.65 mU/g) and blood samples obtained and measured as above. Area under the curve was calculated using Graph Prism.

After 14 weeks on high fat diet, PUMA knockout and wild type mice received two injections (9 am and 5 pm) of recombinant murine leptin (2 μg/g body weight; Peprotech) and body weights and daily food intake were measured during 24 h after the injection.

### Indirect calorimetry measurements

Energy expenditure (EE), respiratory exchange ratio (RER), activity and food intake were assessed using a Comprehensive Lab Animal Monitoring System (CLAMS, Columbus Instruments, USA) after 14–15 weeks on the diet. Body weights were recorded before and after the testing. Mice were acclimatized for 24 h and then monitored for 48 h. The CLAMS is fitted with indirect open circuit calorimetry, activity monitors and scales for food measurements. EE and RER (VCO_2_/VO_2_) were calculated from the gas exchange data. Data was averaged for 2 dark and light cycles.

### Insulin and Leptin measurements

Blood samples of overnight fasted or fed mice were taken, serum prepared and frozen at −80 °C until use. Serum leptin concentrations were determined using a commercial ELISA kit (EZML-82K, Millipore) following manufacturer’s instructions. The glucose-stimulated-insulin-secretion assay was performed as previously described^24^.

### Statistical analysis

Comparisons between groups were made by Student’s *t* test or by ANOVA followed by Bonferroni correction. A p value <0.05 was considered statistically significant.

## Results & Discussion

### PUMA deficiency does not affect body weight gain in high fat diet fed mice

**Figure 1:**
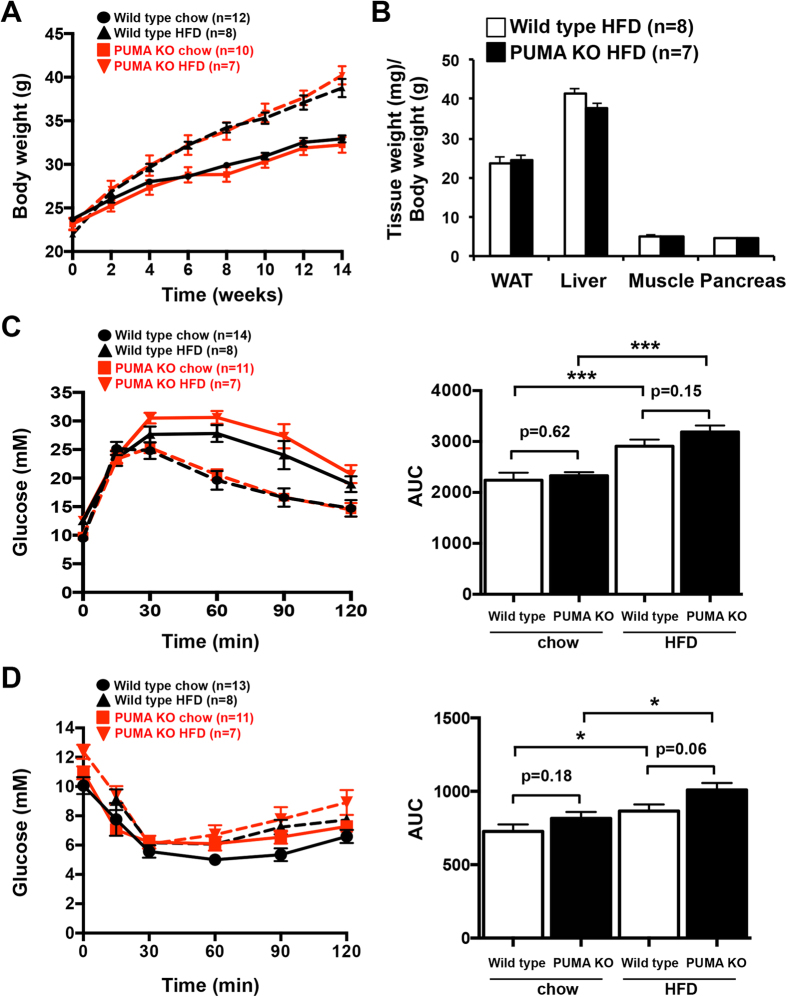
PUMA deficiency does not affect body weight, glucose or insulin tolerance in mice. (**A**) 10 week-old PUMA knockout and wild type male mice were high fat fed for 14 weeks or maintained on a chow diet and body weights determined at the times indicated. (**B**) Body composition (gonadal white adipose tissue (WAT), liver, gastrocnemius muscle and pancreas relative weights) was determined 17 weeks after high fat feeding. (**C**) Glucose tolerance tests (2 mg glucose/g body weight; i.p.) were conducted after 16 weeks of high fat or chow feeding and the areas under the curve (AUC) calculated. (**D**) PUMA deficient and wild type mice after 17 weeks of high fat or chow feeding were subjected to insulin tolerance tests (0.5 mU/g insulin; A.U.C. determined). *p < 0.05, **p < 0.01, ***p < 0.001.

To investigate the impact of PUMA deficiency on body weight changes under obesogenic conditions, we high fat fed PUMA knockout and wild type control animals for 14 weeks. On a regular chow diet, the weight change of PUMA knockout animals was similar to the controls. Male mice on a high fat diet gained on average significantly more body weight that the chow diet as previously reported^23^. However, total body mass gain did not differ between groups (Fig. 1A), in agreement with a previous study^11^.

Next, we compared metabolic tissue weights after chow or high fat feeding. There were no differences in the mass values of adipose tissue, liver, gastrocnemius muscle and pancreas tissue between the PUMA knockout and wild type mice (Fig. 1B; Supp. Fig. 1A). The lack of any effect of PUMA deficiency on total body weight gain under regular or high fat feeding suggests a limited impact of this pro-apoptotic molecule in body weight regulation.

### Blood glucose levels, glucose and insulin tolerance tests and insulin signalling were not affected by PUMA deficiency

PUMA is implicated in apoptosis of β-cells and hepatocytes in conditions of stress that may be experienced in obesity and diabetes, such as high concentrations of circulating fatty acids and/or glucose, and therefore its deficiency may be expected to result in improved metabolism after high fat feeding. In addition, certain BCL-2 family members have been reported to regulate glucose homeostasis. We measured blood glucose levels in 6 h fasted wild type and PUMA knockout mice after 13 weeks on a high fat diet. There was no change in basal blood glucose levels in mice lacking PUMA compared to those observed in control mice (Supp. Fig. 1B). In addition, glucose-stimulated insulin secretion was not affected in isolated PUMA-deficient pancreatic islets (Supp. Fig. 1C).

To assess glucose clearance rates, we performed an i.p. glucose tolerance test. In contrast to mice on a regular chow diet, mice that were on a high fat diet presented a delayed glucose absorbance curve suggesting glucose intolerance. PUMA-deficient mice responded similarly to control mice to the glucose challenge on a chow and high fat diet (Fig. 1C). These results are reflected in the analysis of the area under the curve (AUC) that shows significantly increased values in high fat diet fed mice compared to chow diet fed animals but no differences were detected between the PUMA knockout and wild type animals. Our results reveal that loss of PUMA has no impact on insulin secretion and blood glucose clearance.

To assess the impact of PUMA deficiency on the insulin response, we performed an insulin tolerance test. No significant differences between PUMA knockout and wild type mice were found in glucose level reduction after an i.p. injection of insulin (Fig. 1D).

Next, we studied histology sections and performed gene expression analysis in liver samples from PUMA knockout and wild type mice. As expected, diet-induced obesity triggered fat accumulation in the liver (steatosis) (Supp. Fig. 2A). However, obesity-induced hepatic steatosis was similar between PUMA deficient and wild type mice as assessed by histology (Supp. Fig. 2A). Moreover, no significant differences were observed in gluconeogenic (*g6p, pepck*), lipogenic (*scd1, ppar-γ, aac1*) and inflammatory (*mcp-1, il-6*) gene expression levels in the liver samples (Supp. Fig. 2B).

**Figure 2:**
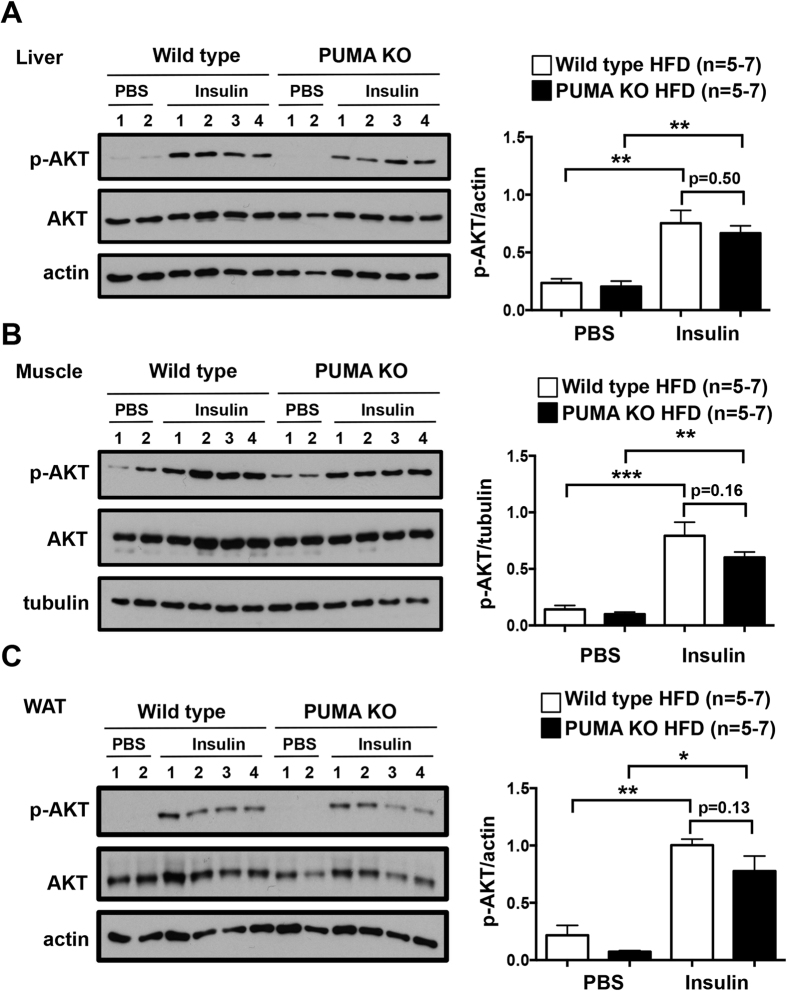
No differences in liver, muscle and adipose tissue insulin sensitivity between high fat fed PUMA knockout and wild type mice. 10 week-old PUMA knockout and wild type male mice were chow-fed or high fat fed for 16 weeks, fasted for 6 h and then injected with PBS or insulin (0.65 mU insulin/g body weight, 10 min). Livers (**A**), muscle (**B**) and abdominal white adipose tissue (**C**) extracted and processed for immunoblotting with the indicated antibodies. The gels have been run under the same experimental conditions and cropped to show protein bands corresponding to p-AKT, AKT, actin or tubulin as indicated. *p < 0.05, **p < 0.01, ***p < 0.001.

To extend our finding that loss of PUMA does not affect whole body glucose homeostasis, we examined insulin receptor signalling in relevant tissues. We measured phosphorylation of AKT in different tissues obtained from high fat fed wild type and PUMA knockout mice exposed to a bolus of insulin. We found a significant increase in the levels of p-AKT in comparison to tissues derived from vehicle-administered animals. However, the level of the response to insulin in liver, muscle and gonadal white adipose tissue of mice lacking PUMA was comparable to that observed in the tissues from wild type controls (Fig. 2A–C). These results reveal that loss of PUMA has no impact on the molecular response to insulin in the liver, white adipose tissue and muscle.

### PUMA deficiency decreases food intake and ambulatory capacity in diet-induced obese mice

**Figure 3:**
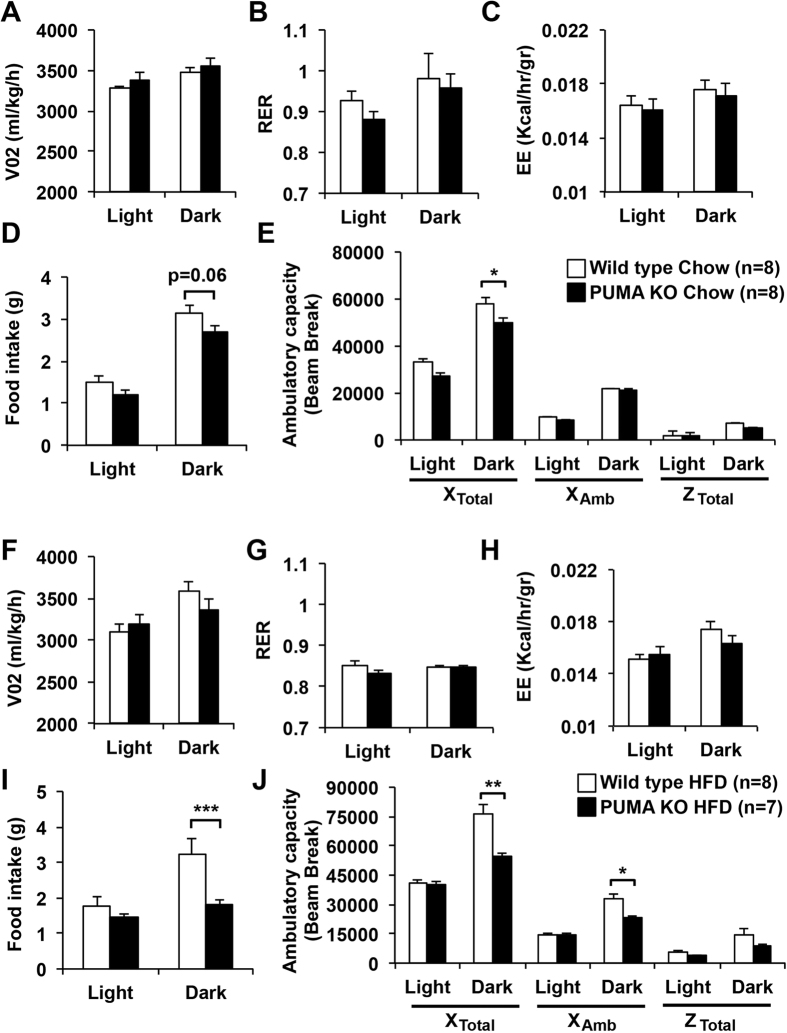
PUMA deficiency decreases food intake and ambulatory capacity in high fat fed mice. Ten-week-old male wild type and PUMA knockout mice were fed a chow (**A–E**) or high fat diet (**F–J**) for 14–15 weeks. Oxygen consumption (VO_2_; **A,F**), respiratory exchange ratios (RER = VO_2_/VCO_2_; **B,G**), energy expenditure (**C,H**), daily food intake (**D,I**) and ambulatory activity (**E,J**) were assessed during the light and dark cycles for 2 consecutive days. *p < 0.05, **p < 0.01, ***p < 0.001.

To evaluate if the absence of PUMA exerts any effect in the whole body metabolic capacity, we measured oxygen consumption, respiratory exchange rate and energy expenditure in chow and high fat fed mice. All these metabolic parameters measured were no different between the PUMA knockout and wild type groups (Fig. 3A–J). Interestingly, we observed that in spite of an equal weight gain between wild type and PUMA knockout mice, the chow fed PUMA deficient animals had decreased ambulatory capacity (Fig. 3E) and a trend towards reduced food intake compared to controls (Fig. 3D). These effects were exacerbated under high fat diet conditions (Fig. 3I,J). The reduced food intake by PUMA knockout mice was probably compensatory to the decreased ambulatory capacity to maintain normal energy expenditure.

### PUMA deficiency increases adipocyte size and fasted leptin levels in high fat fed mice but leptin signalling remains unaffected

**Figure 4:**
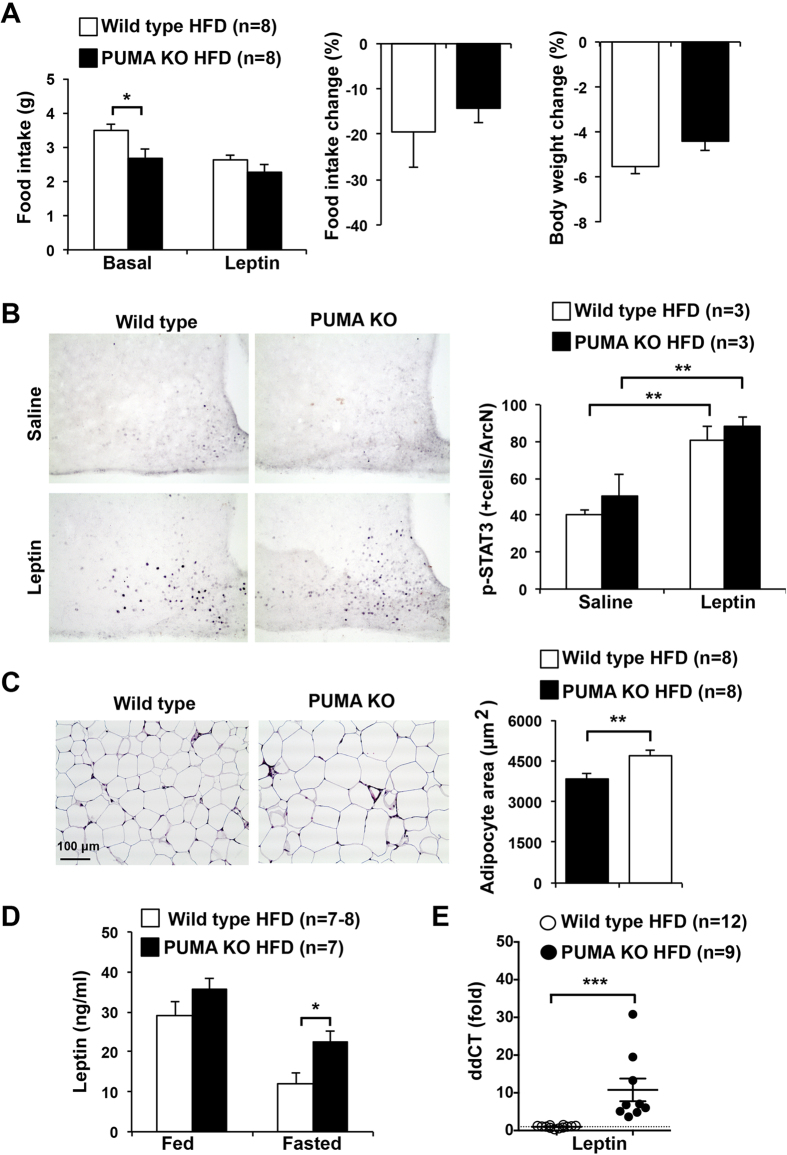
Leptin sensitivity, adipocyte size and leptin levels in high fat fed PUMA knockout and wild type mice. (**A**) 10 week old PUMA knockout and wild type male mice were high fat fed for 14 weeks. Leptin was administered i.p. in the morning and evening and body weight and food intake monitored before and after the treatment. (**B**) 16 week high fat fed mice were fasted for 18 h and injected with saline or leptin and hypothalami extracted and processed for immunohistochemistry with anti-p-STAT3 antibodies. Nuclei positively stained for phosphorylated (i.e. activated) STAT3 in the arcuate nucleus (ArcN) region were counted in serial sections. **p < 0.01. (**C**) Representative haematoxylin and eosin staining of abdominal adipose tissue from 14-week high fat fed PUMA knockout and wild type male mice. Adipocyte area was measured in equal tissue sites from PUMA knockout or wild type mice and averaged values are shown. **p < 0.01. Scale bar is 100 μm. (**D**) Fed and fasted leptin concentration in serum from high fat fed PUMA deficient and wild type mice. *p < 0.05. (**E**) Leptin mRNA expression in white adipose tissue from fasted high fat fed PUMA deficient and wild type mice. The housekeeping gene 18S rRNA was used as normalization control. ***p < 0.001.

Food intake is under control of the hormone leptin, which is a synthesized by adipocytes and acts on the hypothalamus to induce satiety^25^. To directly examine leptin sensitivity, we administered recombinant leptin in the mornings and evenings to a cohort of mice fed a high fat diet for 14 weeks and measured body weights and food intake daily before and after the leptin challenge. We observed reduced food intake in PUMA deficient mice compared to the control animals (Fig. 4A). As expected, leptin administration decreased food intake and body weight in wild type mice. We observed a trend to a reduced impact of leptin on body weight and food intake in the PUMA deficient mice, but this difference was not significant (Fig. 4A).

In the arcuate nucleus of the hypothalamus, leptin acts on anorexigenic, pro-opiomelanocortin (POMC) and orexigenic neuropeptide Y (NPY)- and agouti-related peptide (AgRP)-expressing neurons to decrease food intake^25^. Leptin signals by binding to the leptin receptor (LepRb) to activate the JAK-STAT signalling pathway. We therefore measured STAT3 phosphorylation and activation in neuronal cells after leptin treatment in fasted mice. The high fat fed PUMA knockout and wild type mice showed similar levels of STAT3 activation in the arcuate nucleus, indicating that loss of PUMA has no impact on neuronal leptin signaling (Fig. 4B).

Leptin is mainly produced by adipocytes. We therefore investigated whether decreased food intake in PUMA deficient mice is associated with adipocyte dysfunction. Histologic examination of gonadal white adipose tissue demonstrated that PUMA deficiency increased adipocyte size (Fig. 4C), indicating hypertrophy. This observation was confirmed by quantitative determination of adipocyte diameter by an image analysis system showing an increase in average adipocyte area in high fat fed PUMA knockout mice compared to their wild type counterparts (Fig. 4C). It is well known that leptin secretion is directly correlated with adipocyte size^26^,^27^. Accordingly, we observed increased serum leptin levels in fasted PUMA-deficient mice compared with controls (Fig. 4D). This result was confirmed by measuring the leptin mRNA expression levels in white adipose tissue (Fig. 4E). Increased leptin in the PUMA knockout mice is consistent with the decrease food intake observed in these animals.

To determine if PUMA deletion affects the protein expression of the BH3-only protein BIM, we performed a Western blot analysis in white adipose tissue from high fat fed mice. As shown in Supp. Fig. 3A, PUMA deletion did not change BCL2 or BIM protein expression. Deletion of BH3-only proteins can impact on the immune cells^28^. Thus, we measured IL-1β, IL-6, MCP-1 and IFN-γ expression by qPCR and found decreased levels of IL-1β, IL-6 and MCP-1 in white adipose tissue from PUMA knockout mice (Supp. Fig. 3B), suggesting reduced inflammation.

Obesity induces an enlargement of adipose tissue to store excess energy intake^29^. Adipocyte hypertrophy (increase in cell size) and hyperplasia (increase in cell number) are the mechanisms of fat growth^29^. Hypertrophy occurs prior to hyperplasia to meet the need for additional fat storage capacity in the progression of obesity^29^. Apoptosis of adipocytes is an early event and was reported to affect the development of obesity^14^,^30^. It has been shown that BCL-2 family members, including PUMA, are involved in this process^14^,^15^. Indeed, the expression of the pro-survival protein BCL-2 in adipocytes negatively correlates with BMI^15^. It is conceivable that defects in apoptosis, such as loss of PUMA, allow an abnormal increase in adipocyte size. However, the mechanisms that regulate adipocyte death and expansion are still poorly understood. Thus, further experiments are required to assess the specific roles of the different BCL-2 family members in adipocyte survival and apoptosis and how this may impact on their size and the regulation of leptin secretion. It is notable that adipocyte hypertrophy in high fat fed PUMA deficient mice was not associated with changes in body weight or fat pad mass. This can be explained by previous studies showing that adipocyte size and BMI cannot be adjusted by a linear regression^31^,^32^.

The PUMA knockout mice showed decreased food intake compared to the wild type control mice. It is reasonable to think that a pair-fed wild type group would show reduced body weights, resulting in differences in glucose and insulin responsiveness. Indeed, there is a trend toward higher AUC in the ITT assay and lower p-AKT activity in the high fat fed PUMA knockout mice. Pair feeding will lead to reduced calorie intake and the wild type animals will be calorie restricted (CR). CR is an unsustainable and unviable therapeutic intervention for the vast majority of humans with free access to a wide range of foods^33^.

There are some caveats to our study. We have analysed the impact of PUMA loss on metabolism in mice lacking this pro-apoptotic protein globally. It is therefore not yet established whether PUMA loss has a direct effect in adipocytes and on leptin secretion, or on the immune system affecting fat cells^28^. Thus, tissue specific knockout mouse models will be required^34^ to specifically address the contribution of loss of PUMA to adipoyte hypertrophy and leptin secretion. It is also posible that compensatory effects mediated by a lack of PUMA expression (involving the activity of BCL-2 proteins other than BCL-2 and BIM in white adipose tissue) may impact on the phenotype we observed^35^. Another limitation of our study is that the high fat diet protocol was for 14–17 weeks and may not have been sufficient to highlight long-term changes between the groups. However, this diet increased body weight and induced glucose intolerance as well as insulin resistance. Thus, we are confident that our study appropriately addresses the effects of PUMA deficiency on diet-induced obesity.

Collectively, our data indicate that PUMA loss does not have a marked impact on glucose homeostasis in chow and diet-induced obesity in mice. Interestingly, we observed increased adipocitye size and fasting leptin levels in high fat fed PUMA knockout mice. This correlates with decreased food intake, despite normal neuronal leptin signaling. The decrease in the ambulatory capacity is probably a compensatory effect to maintain normal levels of energy expenditure and body weight. Overall, our study suggests that global inhibition of PUMA does not appear to be a promising approach for the treatment of obesity.

## Additional Information

**How to cite this article**: Litwak, S. A. *et al.* p53-upregulated-modulator-of-apoptosis (PUMA) deficiency affects food intake but does not impact on body weight or glucose homeostasis in diet-induced obesity. *Sci. Rep.* **6**, 23802; doi: 10.1038/srep23802 (2016).

## Supplementary Material

Supplementary Information
