# Supplementary material for: p53-upregulated-modulator-of-apoptosis (PUMA) deficiency affects food intake but does not impact on body weight or glucose homeostasis in diet-induced obesity
Source: Sci Rep. 2016 Apr 1;6:23802. doi: 10.1038/srep23802 (PMC4817123; doi:10.1038/srep23802)
Supplement: Supplementary Information [file srep23802-s1.pdf]

Supplemental Data

p53-upregulated-modulator-of-apoptosis (PUMA) deficiency affects food intake but does not impact on body weight or glucose homeostasis in diet-induced obesity.

Sara A. Litwak, Kim Loh, William J. Stanley, Evan G. Pappas, Jibran A. Wali, Claudia Selck, Andreas Strasser, Helen E. Thomas, Esteban N. Gurzov

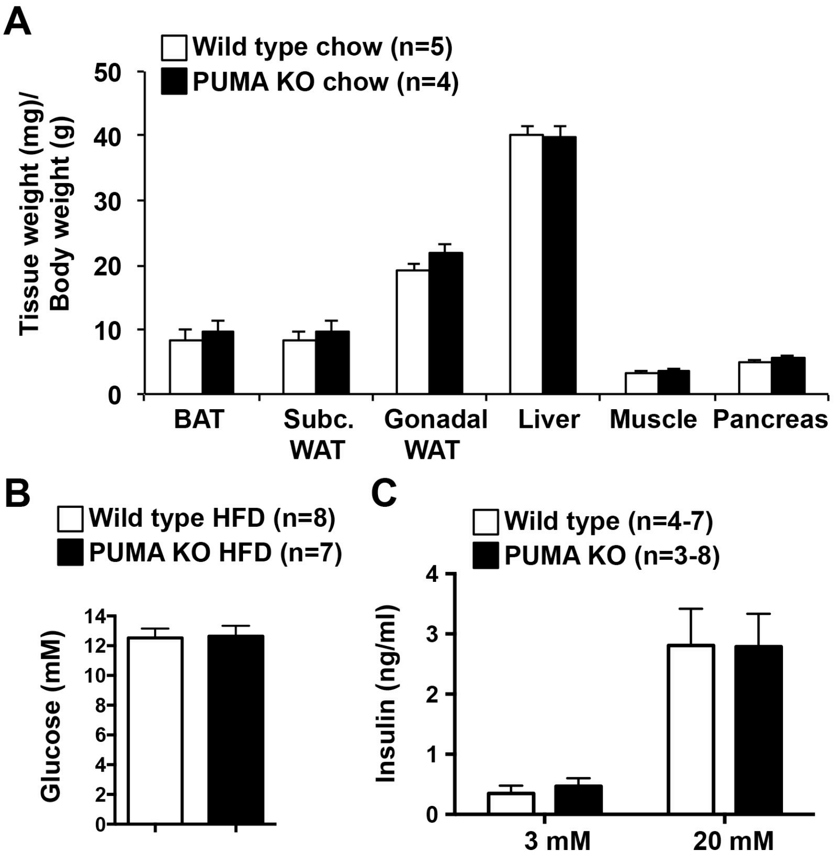

Supplementary Figure 1. A. Body composition (brown adipose tissue (BAT), subcutaneous white adipose tissue (WAT), gonadal WAT, liver, gastrocnemius muscle and pancreas) was determined in chow fed PUMA knockout and wild type mice. B. **8 h** fasted blood glucose in 14 weeks high fat fed PUMA deficient and wild-type male mice. **C.** Islets from PUMA knockout and wild-type male mice were preincubated in Krebs-Ringer buffer for 45 min at 3 mM glucose before being distributed in batches of 10 islets per condition in tubes containing 3 or 20 mM glucose as indicated. Insulin secretion was evaluated after 45 min and corrected for insulin content.

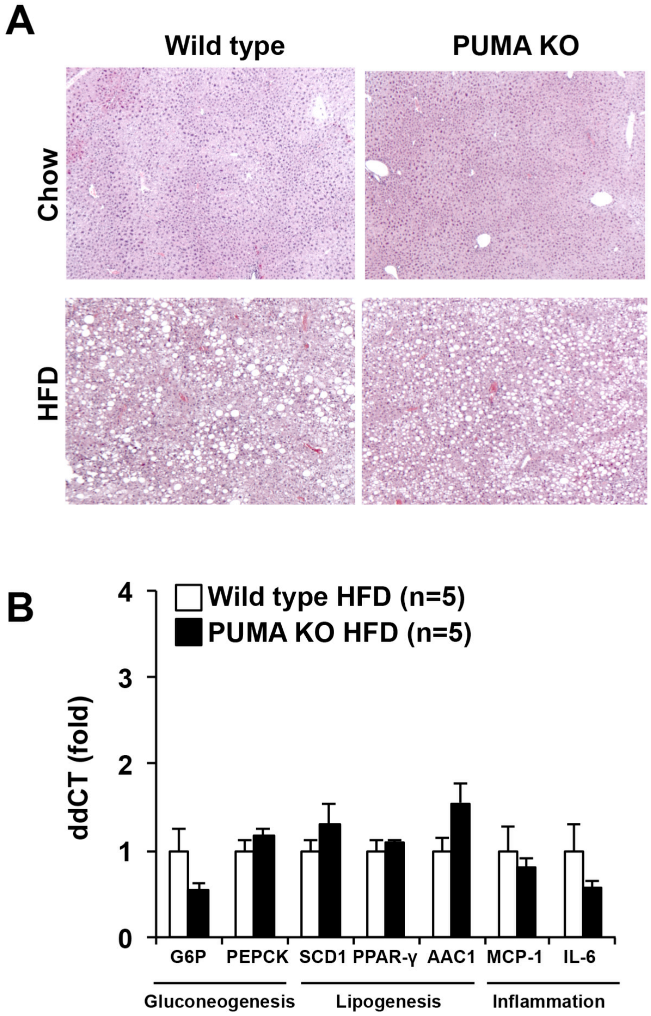

Supplementary Figure 2. A. Livers from 14 week high fat fed PUMA knockout and wild-type male mice were extracted and fixed in formalin and processed for histological assessment staining for haematoxylin & eosin (H&E). **B.** Livers from 14 week high fat fed PUMA knockout and wild-type male mice were processed for quantitative (ddCT) real-time PCR to assess the expression of the gluconeogenic, lipogenic and inflammatory genes as indicated; *actin* was used for normalization.

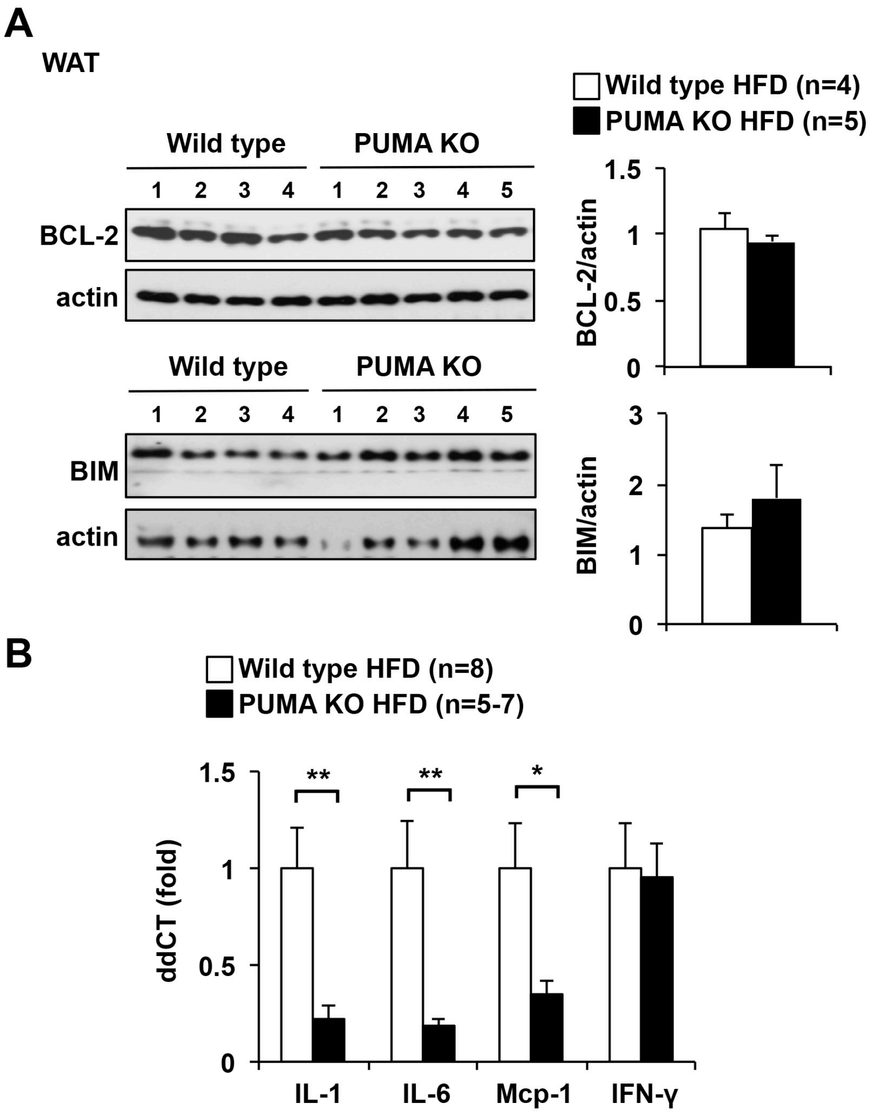

Supplementary Figure 3. A. 10 week-old PUMA knockout and wild type male mice were high fat fed for 16 weeks. Abdominal white adipose tissue extracted and processed for immunoblotting with the antibodies against BIM, BLC-2 or actin. The gels have been run under the same experimental conditions and cropped to show protein bands corresponding to BIM, BCL-2 or actin as indicated. **B.** Abdominal white adipose tissue from 16 week high fat fed PUMA knockout and wild-type male mice were processed for quantitative (ddCT) real-time PCR to assess the expression of the inflammatory genes as indicated; *18s* was used for normalization. *p<0.05, **p<0.01.

Probe	Catalogue number (Applied Biosystems, Foster City, CA, USA)
**G6P (mouse)**	Mm00839363_m1
**PEPCK (mouse)**	Mm00440636_m1
**SCD1 (mouse)**	Mm00772290_m1
**PPARg (mouse)**	Mm01208835_m1
**AAC1 (mouse)**	Mm01304257_m1
**IL-1β (mouse)**	Mm00434228_m1
**IL-6 (mouse)**	Mm00446190_m1
**MCP-1 (mouse)**	Mm00801778_m1
**IFN-γ (mouse)**	Mm00446190_m1
**Leptin (mouse)**	Mm00434759_m1
**Rn18S (mouse)**	Mm003928990_g1
**β-actin (mouse)**	Mm00607939_s1

Supplementary Table 1. List of probes used for qPCR. Real-time PCR was performed using the Rotor-Gene RG-3000 machine (Corbett Research; Qiagen, Hilden, Germany) and the TaqMan PCR Master Mix (AmpliTaq Gold with GeneAmp kit; Applied Biosystems) in 20 µL reaction volumes.
